# Corticosteroid-Dependent Leukocytosis Masks the Predictive Potential of White Blood Cells for Delayed Cerebral Ischemia and Ventriculoperitoneal Shunt Dependency in Aneurysmatic Subarachnoid Hemorrhage

**DOI:** 10.3390/jcm12031006

**Published:** 2023-01-28

**Authors:** Andras Piffko, Franz L. Ricklefs, Nils Schweingruber, Thomas Sauvigny, Marius Marc-Daniel Mader, Malte Mohme, Lasse Dührsen, Manfred Westphal, Jan Regelsberger, Nils Ole Schmidt, Patrick Czorlich

**Affiliations:** 1 Department of Neurosurgery, University Medical Center Hamburg-Eppendorf, 20251 Hamburg, Germany; 2 Department of Radiation and Cellular Oncology, University of Chicago, Chicago, IL 60637, USA; 3 Ludwig Center for Metastasis Research, University of Chicago, Chicago, IL 60637, USA; 4 Department of Neurology, University Medical Center Hamburg-Eppendorf, 20251 Hamburg, Germany; 5 Institute for Stem Cell Biology and Regenerative Medicine, Stanford University School of Medicine, Stanford, CA 94305, USA; 6 Department of Neurosurgery, Diako Hospital Flensburg, 24939 Flensburg, Germany; 7 Department of Neurosurgery, Regensburg University Hospital, 93053 Regensburg, Germany

**Keywords:** subarachnoid hemorrhage, white blood cells, dexamethasone, corticosteroids, outcome, delayed cerebral ischemia, shunt dependency

## Abstract

A multitude of pathological and inflammatory processes determine the clinical course after aneurysmal subarachnoid hemorrhage (aSAH). However, our understanding of predictive factors and therapeutic consequences is limited. We evaluated the predictive value of clinically relevant factors readily available in the ICU setting, such as white blood cell (WBC) count and CRP, for two of the leading comorbidities, delayed cerebral ischemia (DCI) and ventriculoperitoneal (VP) shunt dependency in aSAH patients with and without corticosteroid treatment. We conducted a retrospective analysis of 484 aSAH patients admitted to our institution over an eight-year period. Relevant clinical factors affecting the risk of DCI and VP shunt dependency were identified and included in a multivariate logistic regression model. Overall, 233/484 (48.1%) patients were treated with corticosteroids. Intriguingly, predictive factors associated with the occurrence of DCI differed significantly depending on the corticosteroid treatment status (dexamethasone group: Hunt and Hess grade (*p* = 0.002), endovascular treatment (*p* = 0.016); no-dexamethasone group: acute hydrocephalus (*p* = 0.018), peripheral leukocyte count 7 days post SAH (WBC at day 7) (*p* = 0.009)). Similar disparities were found for VP shunt dependency (dexamethasone group: acute hydrocephalus (*p* = 0.002); no-dexamethasone group: WBC d7 (*p* = 0.036), CRP peak within 72 h (*p* = 0.015)). Our study shows that corticosteroid-induced leukocytosis negates the predictive prognostic potential of systemic inflammatory markers for DCI and VP shunt dependency, which has previously been neglected and should be accounted for in future studies.

## 1. Introduction

Aneurysmal subarachnoid hemorrhage (aSAH) remains a serious life-threatening disease associated with high morbidity and mortality despite advances in neurocritical care, neurosurgical and endovascular treatments [1,2]. Severity of the initial bleeding event determines immediate patient disability and mortality and cannot be therapeutically influenced. However, a multitude of potentially therapeutically targetable complications secondary to aSAH contribute to poor long-term outcomes and generate an extensive health care burden [3,4,5]. One of the leading causes of secondary brain injury and poor neurological outcomes is delayed cerebral ischemia (DCI), which develops in up to one third of patients [5,6,7]. Despite the common misconception that DCI is caused by cerebral vasospasms, less than 50% of patients with angiographic vasospasm actually develop DCI and most studies targeting cerebral vasospasm to prevent cerebral infarction lacked treatment effect, highlighting the need for a better understanding of the pathophysiological processes underlying DCI [7,8]. Aside from the known pathophysiological contributors such as cortical spreading depression and microthromboses, which remain incompletely understood, immune dysregulation has long been thought to attribute to the process of DCI [9,10]. Nonetheless, multiple reports examining immunological changes to predict DCI have shown conflicting results [3,11,12]. Thus, systemic and local inflammation secondary to aSAH remains a controversial and highly complex issue.

Secondary to DCI, hydrocephalus is a major source of morbidity in aSAH patients. Overall, 15–87% of patients develop acute hydrocephalus, and one third of these patients develop the need for constant ventriculoperitoneal CSF drainage [5,13]. Impaired CSF reabsorption, altered CSF dynamics [14,15,16], ependymal inflammation [14,17], amount of ventricular blood [18], and sustained systemic inflammatory response syndrome (SIRS) [16] have proven predictive of chronic hydrocephalus with subsequent ventriculoperitoneal shunt dependency after aSAH.

The role of elevated white blood cell counts (WBC) after aSAH has repeatedly been discussed in the development of DCI as well as of shunt-dependent hydrocephalus [12,16,19]. However, although commonly known to cause leukocytosis and regularly applied in the treatment of microsurgically clipped aSAH patients, most studies do not sufficiently address the role of corticosteroids in the development of secondary complications [13,20,21,22,23]. Nevertheless, corticosteroid application remains a common treatment regimen in neurocritical care, and just this year, a multicenter prospective randomized phase III trial was initiated to test the outcomes and safety of anti-inflammatory treatment with dexamethasone in aSAH patients [24]. In order to improve our understanding of the clinical relevance of systemic inflammation as a prognostic factor for DCI and shunt dependency, we investigated the WBC count, C-reactive protein (CRP) levels, and body temperature within the first 14 days in a large cohort of patients with aSAH while stratifying for the administration of corticosteroids.

## 2. Materials and Methods

All the patients (*n* = 484) admitted between November 2010 to May 2018 with the diagnosis of aSAH were included in this retrospective study. Anonymized data of all the patients was prospectively collected and retrospectively analyzed with approval from the local ethics committee (Ethik-Kommission der Ärztekammer Hamburg, WF-069/18). The study was exempt from the need for informed consent under local law (Hamburger Krankenhausgesetz §12).

To confirm aSAH, either cranial computer tomography (cCT), cranial magnetic resonance imaging (cMRI), or lumbar puncture was performed. Verification of cerebral aneurysms was carried out by means of four-vessel intraarterial three-dimensional rotational digital subtraction angiography (DSA), cCT–angiography, and/or MRI–angiography. Treatment of ruptured aneurysms after interdisciplinary discussion consisted either of surgical clipping or endovascular treatment, performed routinely within 24 h of admission.

Acute hydrocephalus was defined as the need for placement of an external ventricular (EVD) or lumbar drainage (LD) within the first week after admission when patients showed radiological findings of a ventricular dilatation, while shunt dependency was defined as the need for placement of a ventriculoperitoneal shunt system.

Corticosteroid treatment was defined as any administration of at least a single dose of 4 mg dexamethasone in the ICU setting (not including single doses given intraoperatively or under general anesthesia). Until the publication of our previous data [20], corticosteroids were routinely administered in most aSAH patients. Since then, dexamethasone has only been administered to surgically clipped patients at our ICU wards according to our local standard operating procedure. In this patient population, 4 mg dexamethasone is given every 8 h for 5–7 days depending on clinical course, followed by the reduction of the dexamethasone dosage by 50% every two days.

WBC counts were calculated using standardized cell counters and analyzed in routine daily blood draws. The local laboratory reference for the WBC count in adults is 4400 to 11,000 cells/µL and <5 mg/dL for C-reactive protein (CRP). WBC and CRP were measured at least daily for the duration of admission at the intensive care unit. Persistent leukocytosis was defined as a consistent WBC > 11,000 cells/µL throughout d3 and d7, a cut-off value utilized by our institution’s laboratory medicine department. Body temperature was measured continuously using a bladder catheter.

Occurrence of DCI was defined as either (i) clinical deterioration in terms of occurrence of a new focal neurological impairment or a decrease of at least two points on the Glasgow Coma Scale lasting for at least one hour or (ii) presence of cerebral infarction on cranial CT or MRI scans, both not attributable to other causes, including aneurysm occlusion procedures [25].

Outcome was assessed at discharge as well as long-term follow-up by neurosurgical consultants and residents in an outpatient clinic setting and classified according to the modified Rankin Scale score (mRS). The mRS of 0–2 was defined as a favorable outcome, while the mRS of 3–6 was defined as an unfavorable outcome at 6 months.

Statistical analysis of the data was performed by a univariate analysis using a chi-squared test or ANOVA tests depending on the scale of the measurements, using IBM^®^ SPSS^®^ Statistics 22 (IBM Corporation, Armonk, NY, USA) and followed by a multivariable regression analysis. Long-term-follow-up was performed using Cox regression models. The parameters that were significantly associated (*p* ≤ 0.05) with DCI or shunt dependency in each group (no dexamethasone and dexamethasone given) were included in a multivariate regression model. WBC, CRP, and temperature values were extracted from the intensive care unit’s electronic documentation system. The data were processed in a standardized script-based manner with R (version 3.5.3). To visualize the parameters from digital patient records, a regression model (generalized additive model) was fitted over the values.

## 3. Results

### 3.1. Study Cohort

In total, 484 patients were included in this retrospective analysis; 323/484 (66.7%) of the patients were female. The mean age of the cohort was 55.0 ± 13.5 years (range = 18–90 years). Dexamethasone treatment (as described above) was applied in 233/484 (48.1%) patients. The median H&H grade was 3 (range = 1–5), the median GCS was 15 (range = 3–15), and the median Fisher Grade was 4 (range = 1–4).

Out of the 484 patients, 312 (65.0%) developed acute hydrocephalus with the need for external CSF drainage; 144/484 (30.4%) patients presented with intracerebral hemorrhage, and the most commonly applied treatment of ruptured aneurysms was endovascular (314/484 (69.9%)). The secondary complications of interest, (1) DCI and (2) shunt dependency, developed in 180/484 (37.3%) and 65/484 (13.5%) of the cases, respectively. Additional clinical information is provided in Table 1.

**Figure 1:**
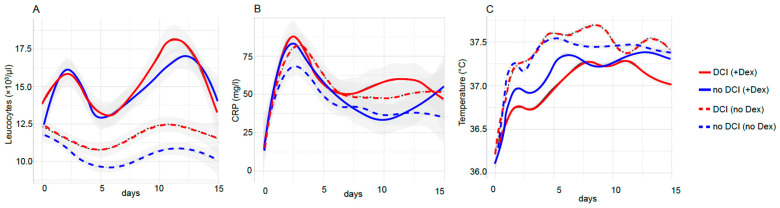
Data of the SAH patients after the first ICU admission was collected for 500 h (approximately 24 days). Depicted are the values of leukocytes (**A**) and C-reactive protein (**B**) in peripheral blood (>8500 values) and the temperature (**C**) (>120,000 measurements using a bladder catheter). The patients who received dexamethasone during their admission to the intensive care unit were considered to belong to the dexamethasone group (continuous line). The SAH patients were further subdivided according to a cohort, which suffered delayed cerebral ischemia. The lines represent a generalized additive model and the confidence interval is in grey. DCI = delayed cerebral ischemia; Dex = dexamethasone; CRP = C-reactive protein.

The clinical course of the mean daily values of the WBC count and serum CRP concentration as well as body temperature (measured continuously using a bladder catheter) were included in a general additive model and depicted for the first 14 days post admission to the ICU (Figure 1). The patients receiving corticosteroid treatment showed significantly higher WBC trends (Figure 1A, Table 2), with two peaks on days 3 and 12 post aSAH. The mean serum CRP values peaked at day 3 post aSAH. The mean body temperature was elevated in all the patient groups and was found to be higher in the patients without corticosteroid treatment. As expected, individual WBC values at admission, d3, d7, and d14, as well as the peak value within the first 72 h and persistent leukocytosis differed significantly between the patients who received steroids and the patients who did not, while there was no significant effect on CRP (Table 2).

As seen in Table 1, despite a slight trend towards higher rates of DCI in the patients who received dexamethasone, this trend did not prove statistically significant (*p* = 0.111), while shunt dependency was significantly associated with dexamethasone treatment (*p* = 0.011) (Table 1). Moreover, the patients receiving dexamethasone had higher H&H grades (*p* = 0.004), higher Fisher grades (*p* = 0.016), and the patients with an intracerebral hemorrhage were more likely to receive steroids (*p* > 0.0001). To account for any biases this may evoke, all following uni- and multivariate analyses were performed independently for the patients who received steroids and those who did not.

### 3.2. White Blood Cell Count and the Impact on Delayed Cerebral Ischemia

Univariate analysis of the patients without dexamethasone treatment revealed that DCI was significantly associated with acute hydrocephalus (*p* = 0.005), WBC count at day 7 (*p* = 0.002) and day 14 (*p* < 0.001), persistent leukocytosis (*p* = 0.016), elevated CRP at day 3 (*p* = 0.038) and day 14 (*p* = 0.012), as well as the Fisher score (*p* = 0.026) (Table S1).

For the patients who received dexamethasone, DCI was associated with the H&H grade (*p* = 0.002), acute hydrocephalus (*p* = 0.002), endovascular treatment (*p* = 0.001), and CRP at day 14 (*p* = 0.031); importantly, all the associations with the WBC count that were observed in the patients without corticosteroid treatment were lost (Table S1).

Multivariate analysis including relevant parameters from the univariate analysis demonstrated that for the patients without dexamethasone, acute hydrocephalus (OR = 2.332, CI = 1.153–4.677, *p* = 0.018) and WBC at day 7 (OR = 1.134, CI = 1.032–1.246, *p* = 0.009) were predictive of the development of DCI. At the same time, for the patients receiving dexamethasone, the Hunt and Hess grade (OR = 1.485, CI = 1.157–1.907, *p* = 0.002) and endovascular treatment (OR = 2.304, CI = 1.169–4.545, *p* = 0.016) were significantly associated with DCI (Table 3 and Table S2).

### 3.3. White Blood Cell Count and the Impact on Ventriculoperitoneal Shunt Dependency

Chronic hydrocephalus requiring constant CSF drainage was present in 13.5% patients (*n* = 64/484). In the patients without dexamethasone treatment, the univariate analysis indicated associations of shunt dependency with the H&H grade (*p* = 0.004), Fisher score (*p* = 0.008), WBC count at day 7 (*p* = 0.005), and CRP value at day 3 (*p* = 0.004) (Table S3). In the patients who received dexamethasone, acute hydrocephalus (*p* = 0.002), intracerebral hemorrhage *(p* = 0.033), and elevated WBC counts at day 3 (*p* = 0.049) showed higher VP shunt necessity rates (Table S3).

Including the significant factors in the multivariate regression analysis, only the WBC count at day 7 (OR = 1.139, CI = 1.008–1.287, *p* = 0.036) and CRP peak within 72 h (OR = 1.007, CI = 1.001–1.013, *p* = 0.015) remained significantly associated with the need for constant CSF drainage in the patients without corticosteroid treatment. When focusing our attention on the patients who received dexamethasone during their ICU stay, the multivariate analysis demonstrated that only acute hydrocephalus (OR = 3.818, CI = 1.410–10.337, *p* = 0.008) was significantly associated with the development of chronic hydrocephalus and the resulting VP shunt dependency (Table 4 and Table S4).

### 3.4. Follow-Up and Outcome

Follow-up and outcome data were available for 332/484 (68.6%) patients at six months. After correcting for the underlying risk factors in a Cox regression model, the parameters known to be associated with unfavorable outcome (e.g., age, H&H) proved to be significant in both patient groups (Table 5). The WBC count on d3 after admission was significantly associated (OR = 1.068, CI = 1.035–1.102, *p* < 0.001) with an unfavorable outcome at 6 months in the whole patient cohort as well as in the patients who received corticosteroid treatment (OR = 1.053, CI = 1.018–1.088, *p* = 0.003). DCI was significantly associated with a worse outcome in the whole cohort and the patients who did not receive corticosteroids (whole-cohort OR = 1.95, CI = 1.386–2.746, *p* < 0.001; no-dexamethasone OR = 2.895, CI = 1.638–5.116, *p* = 0.001; dexamethasone-given OR = 1.441, CI = 0.930–2.232, *p* = 0.102) (Table 5).

## 4. Discussion

There is growing evidence from animal and human studies that demonstrates a positive correlation of leukocytosis with disease severity after aSAH [3,26,27]. Likewise, increasing evidence connects inflammatory mediators such as interleukin 6 (IL-6), CRP, or tumor necrosis factor alpha (TNF-α) with secondary complications such as cerebral vasospasm and DCI [24,28,29], and thus clinical hopes for glucocorticoid treatment with known strong anti-inflammatory effects to potentially positively influence the disease course and prevent secondary complications remain in place [22,24,30]. In this study, we report that although the WBC count can act as a predictive marker for the most commonly observed secondary complications in aSAH patients, this cannot be reliably assessed without simultaneously addressing the administration of corticosteroids. The significant leukocytosis which results from corticosteroid treatment invalidates the WBC counts’ predictive potential.

A hallmark of this study is the stratification of patients into those who received dexamethasone and those who did not. By including this crucial distinction, we found that (1) most patients showed abnormal WBC counts after aSAH, (2) in the patients without dexamethasone treatment, elevated WBC counts at day 7 were associated with the development of DCI as well as the need for permanent CSF drainage, and (3), most importantly, corticosteroid treatment abrogates the predictive value of WBC for both DCI and shunt dependency in aSAH patients.

The administration of corticosteroids in aSAH patients remains controversially discussed, as most available studies are not comparable due to highly variable patient cohorts and corticosteroid medication [20]. However, since recent studies found beneficial effects of corticosteroid administration on neurological long-term follow-up outcomes in patients who underwent microsurgical clipping, we updated our local treatment regimen, now applying corticosteroids only to those patients [20,22].

In recent analyses of the WBC count in aSAH patients, the effect of corticosteroids has mostly been neglected, as also seen in a recent study which utilized the modified Fisher grade together with an elevated WBC count at admission to monitor patients throughout the high-risk phase for DCI [3]. We showed that the administration of corticosteroids clearly induces leukocytosis, a well-known side effect of these drugs [27], which masks the predictive value of the WBC count in the development of DCI and shunt dependency and should thus be accounted for in future studies. In general, DCI was not directly affected by the administration of dexamethasone, which is in line with previous studies [20].

In addition, a recent study by McGirt et al. investigated the role of the peak WBC count as a predictor of vasospasms within the first five days in patients with SAH without stratifying patients by the usage of steroids [12]. Within our longitudinal WBC measurements, we showed that the best separation of WBC counts in patients with and without DCI occurs around day 10, which is most likely too late to actually predict the appearance of cerebral vasospasms or DCI. Therefore, we chose to evaluate WBC counts at multiple points in time and found that elevated WBC counts at day 7 post aSAH were predictive of the development of DCI; however, the causation between cerebral vasospasms, WBC, and DCI cannot be reliably proven. Other studies compared neutrophil percentages in the cerebrospinal fluid of SAH patients and found a relationship to DCI [31,32], again underlining the possibility of an inflammatory pathomechanism for DCI and the importance of further investigating this. It is, furthermore, important to point out that the abovementioned studies partially used multiple differing cutoff values to define leukocytosis.

Approximately 9–64% of aSAH patients develop the need for a constant CSF diversion [15,16,19]. The reasons include impaired CSF reabsorption, ventricular adhesion/obstruction, as well as a systemic inflammatory response syndrome (SIRS) [16]. Preclinical animal studies have demonstrated a hypersecretion of CSF and the resulting CSF accumulation with ventricular expansion as a result of an inflammatory response of the choroid plexus epithelium [14]. Although this study was conducted in a model of intraventricular hemorrhage, it is not uncommon to find intracerebral or -ventricular hemorrhages in patients with aSAH, and recent evidence indicates that the amount of ventricular blood is also associated with a higher rate of ventriculoperitoneal shunting [14,18]. Nevertheless, the impact of elevated WBC counts in terms of the development of shunt dependency is still controversial. Most authors do not stratify their patients according to the administration of dexamethasone, which was also observed in a recent study that showed an elevation of WBC above >14,500 cells/µL blood to be associated with shunt dependency [33]. In our study, we showed that elevated WBC counts at day 7 were associated with the need for constant CSF drainage only in the patients who did not receive dexamethasone, similarly to the predictive value of elevated WBC counts for DCI, whereas the predictive value of WBC is masked by corticosteroid treatment.

Systemic and local inflammation is accepted to play an important and unifying role in the pathogenesis of secondary complications after SAH [34]; however, the role of glucocorticoid treatment remains incompletely understood. A plethora of pro- and anti-inflammatory pathways are known to be initiated in the peri- and intraparenchymal spaces after aSAH. Recent studies have implicated the importance of oxidative stress and bilirubin oxidation, as well as deregulation of the tryptophan–serotonin–kynurenine (TSK) axis that plays a role in initiating inflammatory signaling cascades and might be involved in the pathogenesis of vasospasms, which will be interesting to evaluate in future clinical studies [35,36]. Recent publications showed that microsurgically treated patients receiving corticosteroids showed better long-term neurological outcomes than patients not treated with corticosteroids [20], pointing towards a potential for therapeutic interventions.

Limitations of this study include the monocentric and retrospective nature of our data, which carries the risk of incomplete or incorrect information. Our data were not adjusted for multiple comparisons that were made since we considered the adjustment to be negligible in our data evaluation, and our data only allowed an exploratory analysis and not a confirmatory one. Another potentially significant limitation of our study is the lack of availability of data on clinical or subclinical infections during ICU admission. Bacterial and viral infections are known to increase WBC, CRP, and body temperature and might therefore pose an unknown confounder on the present data. A recent study including 12,299 patients estimated the risk for health care infections (in a point-prevalence survey from US hospitals in 2015) at 3.2% (95% confidence interval 2.9 to 3.5) [37]. However, patients admitted to ICUs are at an inherently higher risk of health care infections due to more commonly necessary indwelling catheters and mechanical ventilation in combination with an increased prevalence of immunosuppression and multidrug-resistant bacteria, which raises the estimate to 15% of patients affected by infections in neurological ICUs [38,39]. In addition to infections as the cause of leukocytosis in aSAH patients, further clinical complications have to be accounted for. For example, the patients who developed the need for external CSF drainage or with an early tracheotomy would be expected to show an intermittent increase in the WBC count in the initial days after surgery. Similarly, the patients who underwent surgical clipping or suffered additional intracerebral or -ventricular hemorrhages might also show temporary leukocytosis and introduce biases. These important factors will be the subject of our future studies and should be accounted for.

Again, reliable numbers of the effects of glucocorticoid treatment in the ICU setting are scarce; however, despite the utmost concern for an increased risk of infections under corticosteroid treatment, a recent meta-analysis of 21 randomized controlled trials (RCTs) in patients with rheumatic diseases has found little to no increased risk of infection in case of short-term application [40,41]. Nevertheless, serious infections during corticosteroid treatment remain a clinical reality, as also suggested in the observational studies analyzed in the aforementioned meta-analysis [9]; this will be analyzed in future studies of our workgroup.

As discussed above, to ultimately draw a conclusion about the administration of steroids in aSAH patients, large multicentric randomized prospective clinical studies are necessary, as currently initiated in Germany (FINISHER trial, α ClinicalTrials.gov (accessed on 30 December 2022) NCT05132920, EudraCT No. 2021-000732-54) [15].

## 5. Conclusions

Leukocytosis independently correlates with DCI and shunt dependency in aSAH patients, although only in patients without accompanying dexamethasone treatment, which has been neglected in previous studies. Our data suggest that inflammatory parameters should only be used to estimate prognosis models if there is no influence from medication such as dexamethasone, as this can bias the results.

## Figures and Tables

**Table 1:** Basic characteristics of the study population.

Characteristic		Overall	No DMX	DMX Given	*p*-Value
		*n* = 484	*n* = 251	*n* = 233	
Gender		323	166	157	0.773
(female)		66.7%	66.1%	67.4%	
Age		55.04 ± 13.51	56.45 ± 13.45	53.52 ± 13.45	0.058
		Range: 18–90	Range: 18–90	Range: 18–86	
Height (cm)		171.94 ± 12.57	172.35 ± 7.89	171.49 ± 16.25	0.546
Weight (kg)		77.01 ± 16.30	76.77 ± 17.20	77.27 ± 15.31	0.759
GCS		15	15	14	0.045
		Range, 3–15	Range, 3–15	Range, 3–15	
Hunt and Hess	1	102 (21.1%)	60 (32.9%)	42 (18.0%)	0.004
	2	139 (28.7%)	82 (32.7%)	57 (42.5%)	
	3	83 (17.1%)	39 (15.5%)	44 (18.9%)	
	4	61 (12.6%)	26 (10.4%)	35 (15.0%)	
	5	99 (20.5%)	44 (17.5%)	55 (23.6%)	
Fisher	1	17 (3.6%)	13 (5.3%)	4 (1.7%)	0.016
	2	47 (9.9%)	22 (8.9%)	25 (10.9%)	
	3	83 (17.4%)	54 (22.0%)	29 (12.6%)	
	4	329 (69.1%)	157 (63.8%)	172 (74.8%)	
Acute hydrocephalus		312 (65.0%)	156 (62.9%)	156 (67.2%)	0.339
Intracerebral hemorrhage		144 (30.5%)	49 (19.5%)	93 (39.9%)	<0.0001
Treatment of aneurysms (endovascular)		314 (69.93%)	187 (80.3%)	127 (58.8%)	<0.0001
DCI		180 (37.3%)	85 (33.9%)	95 (40.9%)	0.111
VP shunt		65 (13.5%)	24 (9.6%)	41 (17.6%)	0.011

DMX = dexamethasone; GCS = Glasgow Coma Scale; DCI = delayed cerebral ischemia.

**Table 2:** White blood cell counts and C-reactive protein values at multiple timepoints.

Parameter		No DMX	DMX Given	*p*-Value
WBC	At admission (*n* = 470)	12.12 ± 4.18	13.31 ± 5.02	0.014
		Range (5–25)	Range (3–29)	
	d3 (*n* = 468)	11.13 ± 3.89	15.18 ± 5.91	<0.0001
		Range (1–25.4)	Range (5.6–40.6)	
	d7 (*n* = 424)	10.42 ± 3.38	13.6 ± 5.18	<0.0001
		Range (2.1–27.1)	Range (1.6–31.7)	
	d14 (*n* = 336)	10.49 ± 4.14	15.74 ± 6.9	<0.0001
		Range (2.1–29.8)	Range (4.8–34.9)	
	Peak within 72 h (*n* = 468)	14.81 ± 4.63	18.63 ± 5.89	<0.0001
		Range (6–31)	Range (7–41)	
Persistent leukocytosis (*n* = 430)		74 (17.2%)	126 (29.3%)	<0.0001
CRP	At admission (*n* = 465)	10.68 ± 19.99	10.45 ± 13.91	0.883
		Range (1.3–167)	Range (1.1–84)	
	d3 (*n* = 468)	72.235 ± 66.39	84.49 ± 75.54	0.083
		Range (5–295)	Range (5–359)	
	d7 (*n* = 423)	44.87 ± 51.33	40.75 ± 58.48	0.441
		Range (5–305)	Range (5–408)	
	d14 (*n* = 337)	32.84 ± 41.16	41.02 ± 50.80	0.279
		Range (5–229)	Range (5–258)	
	Peak within 72 h (*n* = 468)	82.73 ± 70.86	92.47 ± 74.18	0.147
		Range (5–355)	Range (0.22–359)	

CRP = C-reactive protein; DMX = dexamethasone; WBC = white blood cells.

**Table 3:** Multivariate analysis for the development of delayed cerebral ischemia (significant parameters).

No Dexamethasone		
Parameter	Odds Ratio	*p*-Value
	(95% CI)	
Acute hydrocephalus	2.332	0.018
	(1.153–4.677)	
WBC d7	1.134	0.009
	(1.032–1.246)	
Dexamethasone given		
Hunt and Hess	1.485	0.002
	(1.157–1.907)	
Treatment of aneurysms (endovascular)	2.304 (1.169–4.545)	0.016

WBC = white blood cells.

**Table 4:** Multivariate analysis for the development of shunt-dependent hydrocephalus (significant parameters).

No Dexamethasone		
Parameter	Odds Ratio	*p*-Value
	(95% CI)	
WBC d7	1.139	0.036
	(1.008–1.287)	
CRP peak within 72 h	1.007	0.015
	(1.001–1.013)	
Dexamethasone given		
Acute hydrocephalus	3.818	0.008
	(1.410–10.337)	

CRP = C-reactive protein; WBC = white blood cells.

**Table 5:** Cox regression analysis for an unfavorable outcome at 6 months.

Parameter	Whole Cohort (*n* = 332)		No DMX (*n* = 162)		DMX Given (*n* = 170)	
	Odds Ratio	*p*-Value	Odds Ratio	*p*-Value	Odds Ratio	*p*-Value
	(95% CI)		(95% CI)		(95% CI)	
Age	1.033	<0.001	1.071	<0.001	1.019	0.03
	(1.019–1.084)		(1.044–1.099)		(1.002–1.036)	
Hunt and Hess	1.486	<0.001	1.619	<0.001	1.504	<0.001
	(1.295–1.706)		(1.329–1.971)		(1.258–1.798)	
Acute hydrocephalus	1.390	0.240	1.103	0.858	1.473	0.246
	(0.802–2.407)		(0.376–3.235)		(0.766–2.831)	
Shunt dependency	1.099	0.616	1.417	0.273	0.892	0.636
	(0.759–1.592)		(0.760–2.641)		(0.556–1.431)	
WBC d3	1.068	<0.001	1.005	0.91	1.053	0.003
	(1.035–1.102)		(0.924–1.093)		(1.018–1.088)	
Persistent leukocytosis	0.656	0.048	0.640	0.228	0.739	0.259
	(0.432–0.996)		(0.310–1.322)		(0.437–1.249)	
CRP peak within 72 h	1.004	0.001	1.002	0.262	1.004	0.024
	(1.002–1.006)		(0.998–1.006)		(1.000–1.007)	
DCI	1.951	<0.001	2.895	<0.001	1.441	0.102
	(1.386–2.746)		(1.638–5.116)		(0.930–2.232)	
Treatment of aneurysms	1.018	0.927	0.728	0.38	1.186	0.51
(endovascular)	(0.690–1.503)		(0.358–1.478)		(0.714–1.971)	

CRP = C-reactive protein; DCI = delayed cerebral ischemia; DMX = dexamethasone; WBC = white blood cells.

## Data Availability

The data supporting the conclusions of this article will be made available by the authors on reasonable request, without undue reservation (contact via p.czorlich@uke.de).

## Supplementary Materials

The following supporting information can be downloaded at https://www.mdpi.com/article/10.3390/jcm12031006/s1, Table S1: Univariate analysis for delayed cerebral ischemia, Table S2: Multivariate regression for delayed cerebral ischemia, Table S3: Univariate analysis for shunt dependency, Table S4: Multivariate regression for shunt dependency.

## References

[1] van Gijn J., Kerr R.S., Rinkel G.J. (2007). Subarachnoid haemorrhage. Lancet.

[2] Nieuwkamp D.J., Setz L.E., Algra A., Linn F.H., de Rooij N.K., Rinkel G.J. (2009). Changes in case fatality of aneurysmal subarachnoid haemorrhage over time, according to age, sex, and region: A meta-analysis. Lancet Neurol..

[3] Al-Mufti F., Misiolek K.A., Roh D., Alawi A., Bauerschmidt A., Park S., Agarwal S., Meyers P.M., Connolly E.S., Claassen J. (2019). White Blood Cell Count Improves Prediction of Delayed Cerebral Ischemia Following Aneurysmal Subarachnoid Hemorrhage. Neurosurgery.

[4] Bederson J.B., Connolly E.S., Hunt Batjer H., Dacey R.G., Dion J.E., Diringer M.N., Duldner J.E., Harbaugh R.E., Patel A.B., Rosenwasser R.H. (2009). AHA/ASA Guideline Guidelines for the Management of Aneurysmal Subarachnoid Hemorrhage A Statement for Healthcare Professionals From a Special Writing Group of the Stroke Council, American Heart Association. Stroke.

[5] Lawton M., Vates G. (2017). Subarachnoid hemorrhage. N. Engl. J. Med..

[6] Ferguson S., Macdonald R.L. (2007). Predictors of cerebral infarction in patients with aneurysmal subarachnoid hemorrhage. Neurosurgery.

[7] Pluta R.M., Hansen-Schwartz J., Dreier J., Vajkoczy P., Macdonald R.L., Nishizawa S., Kasuya H., Wellman G., Keller E., Zauner A. (2009). Cerebral vasospasm following subarachnoid hemorrhage: Time for a new world of thought. Neurol. Res..

[8] Rowland M.J., Hadjipavlou G., Kelly M., Westbrook J., Pattinson K.T.S. (2012). Delayed cerebral ischaemia after subarachnoid haemorrhage: Looking beyond vasospasm. Br. J. Anaesth..

[9] Cahill J., Zhang J.H. (2009). Subarachnoid Hemorrhage Is It Time for a New Direction?. Stroke.

[10] Macdonald R.L., Schweizer T.A. (2017). Spontaneous subarachnoid haemorrhage. Lancet.

[11] Höllig A., Stoffel-Wagner B., Clusmann H., Veldeman M., Schubert G.A., Coburn M. (2017). Time courses of inflammatory markers after aneurysmal subarachnoid hemorrhage and their possible relevance for future studies. Front. Neurol..

[12] McGirt M.J., Mavropoulos J.C., McGirt L.Y., Alexander M.J., Friedman A.H., Laskowitz D.T., Lynch J.R. (2003). Leukocytosis as an independent risk factor for cerebral vasospasm following aneurysmal subarachnoid hemorrhage. J. Neurosurg..

[13] Connolly E.S., Rabinstein A.A., Carhuapoma J.R., Derdeyn C.P., Dion J., Higashida R.T., Hoh B.L., Kirkness C.J., Naidech A.M., Ogilvy C.S. (2012). Guidelines for the management of aneurysmal subarachnoid hemorrhage: A guideline for healthcare professionals from the american heart association/american stroke association. Stroke.

[14] Karimy J.K., Zhang J., Kurland D.B., Theriault B.C., Duran D., Stokum J.A., Furey C.G., Zhou X., Mansuri M.S., Montejo J. (2017). Inflammation-dependent cerebrospinal fluid hypersecretion by the choroid plexus epithelium in posthemorrhagic hydrocephalus. Nat. Med..

[15] Rincon F., Gordon E., Starke R.M., Buitrago M.M., Fernandez A., Schmidt J.M., Claassen J., Wartenberg K.E., Frontera J., Seder D.B. (2010). Predictors of long-term shunt-dependent hydrocephalus after aneurysmal subarachnoid hemorrhage: Clinical article. J. Neurosurg..

[16] Wessell A.P., Kole M.J., Cannarsa G., Oliver J., Jindal G., Miller T., Gandhi D., Parikh G., Badjatia N., Francois Aldrich E. (2019). A sustained systemic inflammatory response syndrome is associated with shunt-dependent hydrocephalus after aneurysmal subarachnoid hemorrhage. J. Neurosurg..

[17] Li T., Zhang P., Yuan B., Zhao D., Chen Y., Zhang X. (2013). Thrombin-induced TGF-β1 pathway: A cause of communicating hydrocephalus post subarachnoid hemorrhage. Int. J. Mol. Med..

[18] Czorlich P., Ricklefs F., Reitz M., Vettorazzi E., Abboud T., Regelsberger J., Westphal M., Schmidt N.O. (2015). Impact of intraventricular hemorrhage measured by Graeb and LeRoux score on case fatality risk and chronic hydrocephalus in aneurysmal subarachnoid hemorrhage. Acta Neurochir..

[19] Erixon H.O., Sorteberg A., Sorteberg W., Eide P.K. (2014). Predictors of shunt dependency after aneurysmal subarachnoid hemorrhage: Results of a single-center clinical trial. Acta Neurochir..

[20] Czorlich P., Sauvigny T., Ricklefs F., Abboud T., Nierhaus A., Vettorazzi E., Reuter D., Regelsberger J., Westphal M., Schmidt N.O. (2017). Impact of dexamethasone in patients with aneurysmal subarachnoid haemorrhage. Eur. J. Neurol..

[21] Feigin V.L., Anderson N., Rinkel G.J., Algra A., van Gijn J., Bennett D.A. (2005). Corticosteroids for aneurysmal subarachnoid haemorrhage and primary intracerebral haemorrhage. Cochrane Database Syst. Rev..

[22] Gomis P., Graftieaux J.P., Sercombe R., Hettler D., Scherpereel B., Rousseaux P. (2010). Randomized, double-blind, placebo-controlled, pilot trial of high-dose methylprednisolone in aneurysmal subarachnoid hemorrhage. J. Neurosurg..

[23] Mistry A.M., Mistry E.A., Ganesh Kumar N., Froehler M.T., Fusco M.R., Chitale R.V. (2016). Corticosteroids in the management of hyponatremia, hypovolemia, and vasospasm in subarachnoid hemorrhage: A meta-analysis. Cerebrovasc. Dis..

[24] Güresir E., Lampmann T., Bele S., Czabanka M., Czorlich P., Gempt J., Goldbrunner R., Hurth H., Hermann E., Jabbarli R. (2022). Fight INflammation to Improve outcome after aneurysmal Subarachnoid HEmorRhage (FINISHER) trial: Study protocol for a randomized controlled trial. Int. J. Stroke.

[25] Vergouwen M.D.I., Vermeulen M., van Gijn J., Rinkel G.J.E., Wijdicks E.F., Muizelaar J.P., Mendelow A.D., Juvela S., Yonas H., Terbrugge K.G. (2010). Definition of delayed cerebral ischemia after aneurysmal subarachnoid hemorrhage as an outcome event in clinical trials and observational studies: Proposal of a multidisciplinary research group. Stroke.

[26] Miller B.A., Turan N., Chau M., Pradilla G. (2014). Inflammation, vasospasm, and brain injury after subarachnoid hemorrhage. BioMed Res. Int..

[27] Schweingruber N., Fischer H.J., Fischer L., Van Den Brandt J., Karabinskaya A., Labi V., Villunger A., Kretzschmar B., Huppke P., Simons M. (2014). Chemokine-mediated redirection of T cells constitutes a critical mechanism of glucocorticoid therapy in autoimmune CNS responses. Acta Neuropathol..

[28] de Oliveira Manoel A.L., Loch Macdonald R. (2018). Neuroinflammation as a target for intervention in subarachnoid hemorrhage. Front. Neurol..

[29] Saand A.R., Yu F., Chen J., Chou S.H.-Y. (2019). Systemic inflammation in hemorrhagic strokes—A novel neurological sign and therapeutic target?. J. Cereb. Blood Flow Metab..

[30] Mohney N., Williamson C.A., Rothman E., Ball R., Sheehan K.M., Pandey A.S., Fletcher J.J., Jacobs T.L., Thompson B.G., Rajajee V. (2018). A Propensity Score Analysis of the Impact of Dexamethasone Use on Delayed Cerebral Ischemia and Poor Functional Outcomes After Subarachnoid Hemorrhage. World Neurosurg..

[31] Dhar R., Diringer M.N. (2008). The burden of the systemic inflammatory response predicts vasospasm and outcome after subarachnoid hemorrhage. Neurointensive Care.

[32] Tam A.K.H., Ilodigwe D., Mocco J., Mayer S., Kassell N., Ruefenacht D., Schmiedek P., Weidauer S., Pasqualin A., MacDonald R.L. (2010). Impact of systemic inflammatory response syndrome on vasospasm, cerebral infarction, and outcome after subarachnoid hemorrhage: Exploratory analysis of CONSCIOUS-1 database. Neurointensive Care.

[33] Chang S.I., Tsai M.D., Yen D.H.-T., Hsieh C.-T. (2018). The Clinical Predictors of Shunt-Dependent Hydrocephalus Following Aneurysmal Subarachnoid Hemorrhage. Turk. Neurosurg..

[34] Mohme M., Sauvigny T., Mader M.M.D., Schweingruber N., Maire C.L., Rünger A., Ricklefs F., Regelsberger J., Schmidt N.O., Westphal M. (2020). Immune Characterization in Aneurysmal Subarachnoid Hemorrhage Reveals Distinct Monocytic Activation and Chemokine Patterns. Transl. Stroke Res..

[35] Khey K.M.W., Huard A., Mahmoud S.H. (2020). Inflammatory Pathways Following Subarachnoid Hemorrhage. Cell. Mol. Neurobiol..

[36] Saccaro L.F., Pico F., Chadenat M.L., Richard O., Launay J.M., Bastenaire B., Jullien P., Lambert J., Feuga V., Macquet M. (2022). Platelet, Plasma, Urinary Tryptophan-Serotonin-Kynurenine Axis Markers in Hyperacute Brain Ischemia Patients: A Prospective Study. Front. Neurol..

[37] Magill S.S., O’Leary E., Janelle S.J., Thompson D.L., Dumyati G., Nadle J., Wilson L.E., Kainer M.A., Lynfield R., Greissman S. (2018). Changes in Prevalence of Health Care–Associated Infections in U.S. Hospitals. N. Engl. J. Med..

[38] Busl K.M. (2019). Healthcare-Associated Infections in the Neurocritical Care Unit. Curr. Neurol. Neurosci. Rep..

[39] Rosenthal V.D., Maki D.G., Salomao R., Álvarez-Moreno C., Mehta Y., Higuera F., Cuellar L.E., Arikan Ö.A., Abouqal R., Leblebicioglu H. (2006). Device-associated nosocomial infections in 55 intensive care units of 8 developing countries. Ann. Intern. Med..

[40] Dixon W.G., Suissa S., Hudson M. (2011). The association between systemic glucocorticoid therapy and the risk of infection in patients with rheumatoid arthritis: Systematic review and meta- analyses. Arthritis Res. Ther..

[41] Youssef J., Novosad S.A., Winthrop K.L. (2016). Infection Risk and Safety of Corticosteroid Use. Rheum. Dis. Clin. N. Am..

