# Supplementary material for: Corticosteroid-Dependent Leukocytosis Masks the Predictive Potential of White Blood Cells for Delayed Cerebral Ischemia and Ventriculoperitoneal Shunt Dependency in Aneurysmatic Subarachnoid Hemorrhage
Source: J Clin Med. 2023 Jan 28;12(3):1006. doi: 10.3390/jcm12031006 (PMC9917511; doi:10.3390/jcm12031006)
Supplement: Supplementary file 1 [file jcm-12-01006-s001.zip › jcm-2163782-supplementary.pdf]

Univariate Analysis for Delayed cerebral ischemia

Parameter		No Dexamethasone			Dexamethasone given		
		DCI	no DCI	p	DCI	no DCI	p
**Age (n=251)**		55.97 ± 12.02	56.71 ± 14.16	0.120	52.75 ± 13.93	54.05 ± 13.18	0.47
**Gender (female) (n=251)**		28 (32.9%)	57 (34.3%)	0.889	32 (33.7%)	43 (31.4%)	0.776
**Hunt & Hess (n=251)**		2 (range 1-5)	2 (range 1-5)	0.626	4 (range 1-5)	3 (range 1-5)	**0.002***
**Fisher (n=246)**		4 (range 1-4)	4 (range 1-4)	**0.026***	4 (range 1-4)	4 (range 1-4)	0.069
**Acute Hydrocephalus (n=248)**		63 (75.0%)	93 (56.7%)	**0.005***	80 (58.8%)	75 (78.9%)	**0.002***
**Intracerebral Hemorrhage (n=243)**		19 (23.5%)	30 (18.5%)	0.398	35 (38.7%)	58 (42.6%)	0.768
**Treatment of Aneurysm (Endovascular) (n=233)**		62 (74.7%)	125 (83.3%)	0.124	66 (71.7%)	60 (48.8%)	**0.001***
**WBC**	**at admission (n=239)**	12.40 ± 4.59	11.98 ± 3.98	0.468	13.79 ± 5.20	12.92 ± 4.85	0.199
	**d3 (n=233)**	11.59 ± 3.91	10.88 ± 3.87	0.186	15.95 ± 5.67	15.68 ± 6.09	0.74
	**d7 (n=217)**	11.47 ± 3.98	9.81 ± 2.81	**0.002***	13.53 ± 4.89	13.56 ± 5.36	0.958
	**d14 (n=174)**	11.88 ± 4.68	9.58 ± 3.48	**<0.001***	16.53 ± 7.05	15.08 ± 6.82	0.186
	**peak within 72h (n=233)**	15.68 ± 4.46	14.38 ± 4.67	**0.04***	19.19 ± 5.83	18.19 ± 5.89	0.21
**Persistent Leukocytosis (n=217)**		34 (44.2%)	40 (27.4%)	**0.016***	55 (61.8%)	70 (59.8%)	0.886
**CRP**	**at admission (n=240)**	13.24 ± 26.89	9.40 ± 15.39	0.161	11.06 ± 14.72	10.07 ± 13.42	0.604
	**d3 (n=234)**	84.61 ± 71.13	65.68 ± 63.01	**0.038***	85.19 ± 75.59	83.48 ± 75.87	0.869
	**d7 (n=218)**	50.97 ± 53.13	41.40 ± 50.15	0.187	42.85 ± 60.09	38.36 ± 57.12	0.586
	**d14 (n=174)**	42.40 ± 50.89	26.41 ± 31.74	**0.012***	49.52 ± 56.67	32.47 ± 42.79	**0.031***
	**peak within 72h (n=234)**	95.72 ± 71.94	76.07 ± 69.59	**0.041***	99.37 ± 73.71	87.33 ± 74.46	0.234

Supplement Table S1

Multivariate Regression for Delayed Cerebral Ischemia

No Dexamethasone

Parameter	Odds ratio (95% CI)	p-value
**Fisher**	1.174 (0.740-1.861)	0.495
**WBC peak within 72h**	0.986 (0.907-1.071)	0.986
**Persistent Leukocytosis**	1.894 (1.033-3.475)	0.569
**CRP d3**	1.003 (0.999-1.008)	0.16
**CRP peak within 72h**	0.995 (0.982-1.008)	0.445

Dexamethasone given

Parameter	Odds ratio (95% CI)	p-value
**Acute Hydrocephalus**	1.338 (0.57-3.139)	0.504
**CRP d14**	1.005 (0.998 -1.012)	0.136

Supplement Table S2

Univariate Analysis for Shunt dependency

Parameter		No Dexamethasone			Dexamethasone given		
		Shunt	no Shunt	p	Shunt	no Shunt	p
**Age (n=250)**		60.13 ± 13.47	56.01 ± 13.42	0.154	55.88 ± 13.95	53.01 ± 13.33	0.217
**Gender (female) (n=250)**		7 (29.2%)	77 (34.1%)	0.821	10 (24.4%)	66 (34.4%)	0.272
**Hunt & Hess (n=251)**		4 (range 1-5)	2 (range 1-5)	**0.004***	3 (range 1-5)	3 (range 1-5)	0.266
**Fisher (n=246)**		4 (range 1-4)	4 (range 1-4)	**0.008***	4 (range 1-4)	4 (range 1-4)	0.078
**Acute Hydrocephalus (n=247)**		24 (100.0%)	131 (58.7%)	**<0.0001***	36 (87.8%)	120 (62.8%)	**0.002***
**Intracerebral Hemorrhage (n=242)**		7 (30.4%)	42 (19.2%)	0.272	24 (56.5%)	70 (37.7%)	**0.033***
**Treatment of Aneurysm (Endovascular) (n=232)**		19 (79.2%)	167 (80.3%)	1	25 (61.0%)	102 (58.3%)	**0.86**
**WBC**	**at admission (n=238)**	12.79 ± 4.59	12.07 ± 4.15	0.445	14.50 ± 4.72	13.06 ± 4.72	0.341
	**d3 (n=232)**	12.10 ± 3.29	11.03 ± 3.96	0.213	17.48 ± 7.02	15.45 ± 5.60	**0.049***
	**d7 (n=216)**	12.76 ± 4.71	10.13 ± 3.07	**0.005***	14.58 ± 5.41	13.36 ± 5.11	0.183
	**d14 (n=173)**	11.95 ± 3.01	10.27 ± 4.27	0.67	17.27 ± 6.75	15.27 ± 6.95	0.12
	**peak within 72h (n=232)**	15.90 ± 4.40	14.7 ± 4.66	0.243	19.95 ± 6.61	18.36 ± 5.70	0.125
**Persistent Leukocytosis (n=216)**		14 (60.9%)	60 (30.2%)	**0.005***	26 (66.7%)	100 (59.5%)	0.469
**CRP**	**at admission (n=239)**	24.81 ± 39.49	9.22 ± 16.37	0.068	11.36 ± 15.17	10.27 ± 13.68	0.665
	**d3 (n=233)**	109.44 ± 69.19	67.55 ± 64.48	**0.004***	99.18 ± 81.54	81.44 ± 74.11	0.188
	**d7 (n=217)**	55.81 ± 52.94	43.61 ± 51.23	0.274	48.59 ± 51.94	38.91 ± 59.91	0.353
	**d14 (n=173)**	43.83 ± 41.35	31.05 ± 41.14	0.16	43.00 ± 51.87	40.44 ± 50.68	0.788
	**peak within 72h (n=233)**	133.74 ± 70.50	76.80 ± 68.55	**<0.0001***	108.47 ± 76.12	89.24 ± 73.57	0.145

Supplement Table S3

Multivariate Regression for Shunt dependency

No Dexamethasone

Parameter	Odds ratio (95% CI)	p-value
**Hunt & Hess**	1.326 (0.889-1.978)	0.167
**Fisher score**	2.071 (0.689-6.225)	0.173
**Acute Hydrocephalus**	not computable†	
**Persistent Leukocytosis**	0.952 (0.226-4.007)	0.942
**CRP d3**	0.993 (0.997-1.010)	0.433

† all patients requiring a shunt had an acute hydrocephalus, therefore it is impossible to include this parameter in a multivariate regression model, see supplement Table 3

Dexamethasone given

Parameter	Odds ratio (95% CI)	p-value
**Intracerebral Hemorrhage**	2.412 (1.175-4.953)	0.16
**WBC d3**	1.049	0.115

Supplement Table S4
